# SUBJECTIVE MEMORY COMPLAINTS AND COGNITIVE PERFORMANCE: THE MODERATING ROLE OF DEPRESSIVE SYMPTOMS

**DOI:** 10.1093/geroni/igz038.2425

**Published:** 2019-11-08

**Authors:** Elizabeth A Gallagher

**Affiliations:** University of Massachusetts Boston, Boston, Massachusetts, United States

## Abstract

Cognitive health is a rising public health concern in the U.S. Currently, approximately 5.7 million older adults suffer from Alzheimer’s disease (AD), and by the year 2050 this number is expected to increase to 14 million. Subjective memory complaints (SMC) are shown to be an early indicator of cognitive decline, and accordingly included as a clinical criterion for diagnoses of MCI, an indicator of pre-dementia states, and a research criterion for AD diagnoses. Among older adults, depressive symptoms hinder the accuracy of memory self-ratings. However, there has yet to be consensus regarding the nature of how depressive symptoms may condition the relationship between SMC and cognitive performance. The aims of the present study are to both investigate whether SMC is related to episodic memory and to determine whether depressive symptoms act as a moderator for the relationship between SMC and episodic memory among older adults. This research used nationally representative sample of 8,123 older adults aged 65 and older who completed the Leave Behind Questionnaire in the 2012 and 2014 waves of the Health and Retirement Study. Linear regression was performed and results showed that there was a significant main effect of SMC on episodic memory performance, in that older adults with increased SMC have worse episodic memory. There was also a significant moderating effect of depressive symptoms, in that depressive symptoms cause older adults to underestimate their memory abilities. In order to use SMC as a tool for early detection efforts it is critical to understand these complex relationships.

